# All-optical forward-viewing photoacoustic probe for high-resolution 3D endoscopy

**DOI:** 10.1038/s41377-018-0070-5

**Published:** 2018-10-10

**Authors:** Rehman Ansari, Edward Z. Zhang, Adrien E. Desjardins, Paul C. Beard

**Affiliations:** 1 Department Medical Physics and Biomedical Engineering, University College London, Gower Street, London, WC1E 6BT UK; 2 Wellcome/EPSRC Centre for Interventional and Surgical Sciences, University College London, Charles Bell House, 67-73 Riding House Street, London, W1W 7EJ UK

## Abstract

A miniature forward-viewing endoscopic probe that provides high-resolution 3D photoacoustic images is demonstrated. The probe is of outer diameter 3.2 mm and comprised of a transparent Fabry-Pérot (FP) polymer-film ultrasound sensor that is located at the distal end of a rigid optical fiber bundle. Excitation laser pulses are coupled simultaneously into all cores of the bundle and are transmitted through the FP sensor to provide wide-field tissue illumination at the distal end. The resulting photoacoustic waves are mapped in 2D by sequentially scanning the input end of the bundle with an interrogation laser beam in order to individually address different points on the FP sensor. In this way, the sensor acts as a high-density ultrasound array that is comprised of 50,000 individual elements, each of which is 12 µm in diameter, within the 3.2 mm diameter footprint of the probe. The fine spatial sampling that this affords, along with the wide bandwidth (*f*_-3dB = _34 MHz) of the sensor, enables a high-resolution photoacoustic image to be reconstructed. The measured on-axis lateral resolution of the probe was depth-dependent and ranged from 45-170 µm for depths between 1 and 7 mm, and the vertical resolution was 31 µm over the same depth range. The system was evaluated by acquiring 3D images of absorbing phantoms and the microvascular anatomies of a duck embryo and mouse skin. Excellent image fidelity was demonstrated. It is anticipated that this type of probe could find application as a tool for guiding laparoscopic procedures, fetal surgery and other minimally invasive interventions that require a millimeter-scale forward-viewing 3D photoacoustic imaging probe.

## Photoacoustic imaging: Looking forward to miniaturized endoscopy

Doctors looking for minimally invasive imaging techniques for use during procedures such as colon cancer screening may soon use laser-generated ultrasound waves from photoacoustic devices. Researchers are increasingly trying to equip endoscopes, slender visualization and diagnostic instruments inserted into the body, with photoacoustics because ultrasound penetrates deeper into tissue than light. Paul Beard from University College London in the United Kingdom and colleagues have now developed the first photoacoustic endoscope that images in the same forward direction of endoscope travel. By capping multi-core fiber-optic cables with thin, transparent polymer films that sense ultrasound waves, the team’s approach avoids the need for separate and bulky detectors. A prototype device consisting of 50 000 individually addressable fiber-optic cores produced high-resolution images of microvascular network of samples including duck embryos and mouse skin.

## Introduction

Photoacoustic imaging^1–4^ employs laser-generated ultrasound to provide images of absorbing soft-tissue structures. It provides greater penetration depths than purely optical imaging methods that rely on ballistic photons, such as light microscopy and optical coherence tomography (OCT), and yields higher resolution than diffuse optical methods. It offers a combination of selective optical absorption contrast, high spatial resolution and deep penetration, thereby enabling detailed visualization of tissue types based on endogenous hemoglobin, lipid, melanin, and other chromophores.

To date, substantial effort in clinical photoacoustic imaging^5,6^ has been focused on the development of non-invasive imaging instruments for applications such as the assessment of breast cancer^7^, Crohn’s disease^8^, metastatic lymph nodes^9^, skin cancer^10^, and dermatological conditions^11,12^. However, there is increasing interest in developing miniature endoscopic photoacoustic devices for minimally invasive applications such as the assessment of prostate and colorectal cancers and coronary artery disease and guiding interventional procedures such as laparoscopic surgery. Realizing suitably miniature devices is challenging. Most previously reported photoacoustic endoscopic devices^13–21^ are side-viewing probes that comprise a single ultrasound receiver located at the distal end of an optical fiber that emits laser pulses orthogonal to its axis. The probe is mechanically rotated, thereby allowing the visualization of the interior of cylindrical anatomical structures such as the coronary arteries. However, a miniaturized forward-viewing probe that can “look ahead” in the direction of insertion is desirable for many applications, including guiding minimally invasive interventional procedures such as laser therapy and needle biopsy to organs such as the placenta^22^, pancreas^23^, and liver^24^. Forward-viewing requires lateral scanning of the ultrasound detector; however, this is significantly more challenging to achieve than the rotational motion of a side-viewing probe, as it requires a highly miniaturized x–y scanner to be integrated at the probe tip. An array of ultrasound receivers could be used as an alternative. However, most conventional ultrasound detectors are opaque. Therefore, the array would need to be offset from the delivery optical fiber to avoid obscuring the excitation light, thereby increasing the probe dimensions. Furthermore, achieving sufficiently fine spatial sampling for high-resolution 3D endoscopic imaging requires an extremely high-density 2D array that is composed of several tens of thousands of wideband (tens of MHz) detector elements arranged within the millimeter-scale footprint of the probe. Fabricating such an array with sufficient sensitivity and bandwidth using current conventional piezoelectric or capacitive micromachined ultrasonic transducers (CMUT) detection methods represents a major challenge. For these reasons, a forward-viewing 3D photoacoustic imaging endoscopic probe that is based on either single-detector scanning or a 2D array has not been demonstrated previously.

In this paper, we describe an approach that addresses these limitations, thereby enabling the first demonstration of a miniature forward-viewing 3D photoacoustic probe. The probe comprises a coherent fiber bundle with a Fabry-Pérot (FP) polymer-film ultrasound sensor at its distal end; while this type of sensor has been used in free-space non-invasive photoacoustic scanners^11,25,26^, it has not previously been demonstrated in an endoscopic photoacoustic imaging device. A key advantage is that the sensor is transparent; hence, in contrast to opaque piezoelectric receivers, there is no need to laterally offset it from the excitation beam. This enables the realization of a forward-viewing configuration in which the probe dimensions are limited only by the dimensions of the bundle, thereby enabling a small footprint to be achieved. In this study, we demonstrate a probe of outer diameter 3.2 mm, which is sufficiently small to insert through the working channel of an endoscope. Moreover, each point on the FP sensor is individually addressable via the individual cores of the fiber bundle. Since the packing density of a coherent fiber bundle is extremely high ( > 6000 cores/mm^2^), this means the sensor acts as an ultra-high-density ultrasound array that is composed of several tens of thousands of micron-scale detector elements located within a millimeter-scale footprint. This is consistent with the level of spatial sampling that is required for high-resolution endoscopic photoacoustic imaging but unattainable using conventional ultrasound detection technology.

In this paper, we demonstrate the feasibility of interrogating the FP sensor at the tip of a fiber bundle to realize a 3D forward-viewing photoacoustic imaging probe that has the potential for endoscopic use. Compared to free-space implementations of the FP sensor concept^25^, this required addressing several critical challenges. These include scanning the input end of the bundle with sufficient precision to couple light into individual fiber cores and compensating for the non-ideal light propagation characteristics of the fiber bundle to optimally interrogate the sensor and minimize noise. The experimental set-up and the sensor interrogation and read-out scheme are described below, followed by an assessment of the imaging performance of the probe using phantoms and biological tissues.

## Materials and methods

### Experimental arrangement

**Fig. 1:**
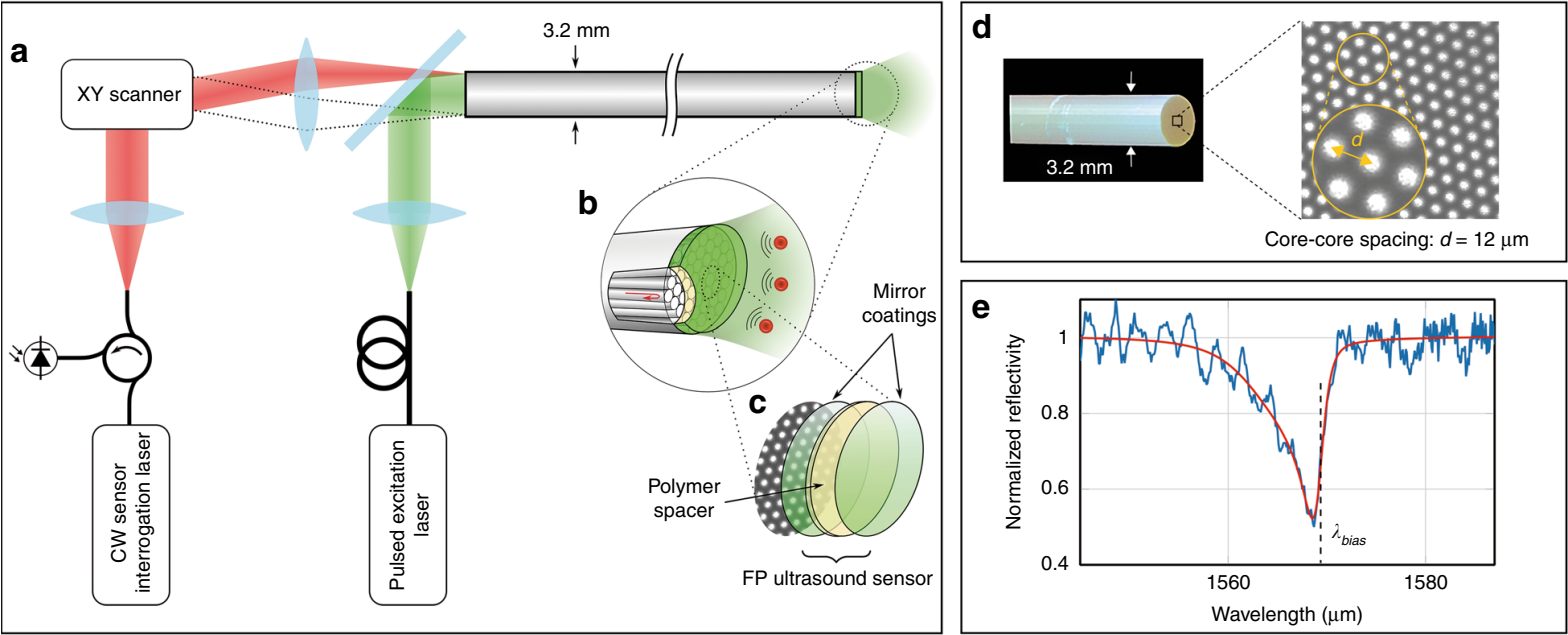
All-optical forward-viewing photoacoustic endoscopy probe. **a** A schematic representation of the probe. **b** A magnified visualization of the distal end shows individual fiber-optic cores in the coherent fiber bundle and the Fabry-Pérot (FP) ultrasound sensor. **c** The FP sensor is deposited on the distal end of the fiber bundle and is comprised of two dielectric mirror coatings that are sandwiched between a Parylene C spacer layer (15 µm thick)—see also Supplementary Fig. S1. **d** A bright-field microscope image of the tip of the fiber bundle (prior to deposition of the FP sensor), which shows a subset of the 50,000 fiber cores. Suppl. Fig. S2 shows an image of the entire bundle, which illustrates all 50,000 cores and was obtained by scanning the interrogation beam over the proximal end of the bundle with the FP sensor in situ. **e** The interferometer transfer function (ITF) from a single core position. Blue: the measured ITF. Red: an asymmetric Lorentzian fit to the measured ITF

A schematic diagram of the experimental arrangement that is used to demonstrate the concept is shown in Fig. 1a. In contrast to the scanning microscopy modes that are most often employed in minimally invasive photoacoustic devices^14–19^, the probe operates in 3D tomography mode. This involves using wide-field illumination to irradiate a relatively large volume of tissue and recording the acoustic waves in 2D at the tip of the probe.

The probe consists of a rigid 76 mm long, 3.2 mm diameter fiber-optic bundle (Edmund Optics, Inc.) that is comprised of 50,000 cores, each of 12 µm diameter and a 12 µm core-to-core spacing, with a transparent FP polymer-film ultrasound sensor deposited on its distal end-face (Fig. 1b, c—see also Suppl. Fig. S1). The very high-density of the cores, each of which can be regarded as an individually addressable ultrasound receiver, is illustrated in Fig. 1d and Suppl. Fig. S2. The proximal end of the bundle was polished to form an 8 degree angle (not shown) to minimize parasitic interference between the light that is reflected from the two ends of the bundle, which can otherwise introduce noise. The FP sensor was formed by vacuum depositing a 15 µm thick Parylene C film layer between two 90% reflective dielectric mirrors, followed by the deposition of a 5 µm thick protective layer of Parylene C to prevent water ingress^25^. The dielectric mirrors are designed to be dichroic and transparent to light in the 580–1250 nm spectral range to permit transmission of the photoacoustic excitation laser pulses through the sensor, but highly reflective (90%) between 1400 and 1600 nm, which coincides with the wavelength range of the interrogation laser used to read-out the sensor.

To generate the photoacoustic waves, two excitation laser sources were used variously. For the tissue phantom studies, a fiber-coupled Q-switched Nd:YAG laser that was emitting 7 ns pulses at 1064 nm with a pulse repetition frequency (PRF) of 20 Hz was used. To acquire images in biological tissues, a tunable (410–2100 nm) fiber-coupled optical parametric oscillator (OPO)-based laser system (Innolas Spitlight 600) that was emitting 7 ns pulses at a PRF of 30 Hz was used. The excitation laser beam is expanded to fill the entire aperture of the proximal end of the bundle and emerges at the distal end, where it is transmitted through the sensor and into the target tissue, thereby providing wide-field illumination. Absorption of the laser energy generates acoustic waves that propagate back to the sensor, where they modulate its optical thickness and, thus, its reflectivity (Suppl. Fig. S1). Then, the sensor is read-out point by point by scanning a continuous-wave, focused interrogation laser beam from core to core at the proximal end of the bundle using a precision x–y galvanometer-based scanner designed to provide sub-micron positional accuracy; Suppl. Fig. S3 illustrates the pointing stability and repeatability of the scanner. The interrogation laser was an external-cavity laser (Tunics T100s-HP, Yenista Optics) that provides a continuous-wave output with a linewidth of 100 kHz, tuning range 1500–1630 nm and an output power between 6 and 20 mW, depending on wavelength. The light that is reflected from each FP sensor location is directed via a single-mode fiber-optic circulator on to an InGaAs photodiode-amplifier unit that is connected to a 250 MS/s digitizer with a 125 MHz analog bandwidth. Optically addressing the FP sensor with a fiber bundle in this way effectively synthesizes a high-density 2D ultrasound array that is composed of 50,000 detector elements, each with an element diameter that is determined by the fiber core diameter (12 µm) and an inter-element spacing that is determined by the core-to-core spacing (12 µm) (see Suppl. Fig. S2).

The time that is required to scan the bundle and acquire the photoacoustic signals depends on four factors: (i) the galvanometer small-step response time: 0.1 ms; (ii) the tuning speed of the interrogation laser: 150 ms for small steps ( < 1 nm); (iii) the number of times the interrogation laser must be tuned during the scan: ~15 in total since many cores have the same bias wavelength; and (iv) the PRF of the excitation laser: 20 Hz or 30 Hz, depending on which excitation laser was used. Of these four factors, the excitation laser PRF dominates the total image acquisition time.

### FP sensor interrogation scheme

To achieve maximum acoustic sensitivity, it is necessary to optimally bias the FP sensor. This requires tuning the interrogation laser wavelength to the point of maximum slope on the FP interferometer transfer function (ITF), which represents the relationship between the reflected optical power and wavelength^25^. Under these conditions, an incident acoustic wave modulates the optical thickness of the polymer spacer, thereby producing a corresponding modulation in the reflected power. An example ITF that was obtained by interrogating the FP sensor through a single core of the bundle is shown in Fig. 1e. The asymmetric shape of the ITF reflectance minimum is due to the accumulation of the Gouy phase shift by the Gaussian interrogation beam over several round trips in the FP cavity^27,28^. In addition to the primary reflectance minimum, high-frequency parasitic oscillations are observed. These are due to crosstalk and a consequence of the very tightly packed nature of the cores, which results in wavelength-dependent coupling between adjacent cores. These oscillations distort the ITF, thereby making it difficult to accurately identify the optimum bias wavelength (*λ*_bias_). To mitigate this and acquire an accurate representation of the ITF, an asymmetric Lorentzian function was fitted (shown as the red line in Fig. 1e) to the measured ITF. Then, *λ*_bias_ is determined by taking the derivative of the fitted curve. To spatially map the photoacoustic waves that arrive at the FP sensor, the proximal end of the bundle is scanned in 2D with each core being addressed at its λ_bias_ by appropriate adjustment of the interrogation laser wavelength^29^.

### FP sensor acoustic performance

**Fig. 2:**
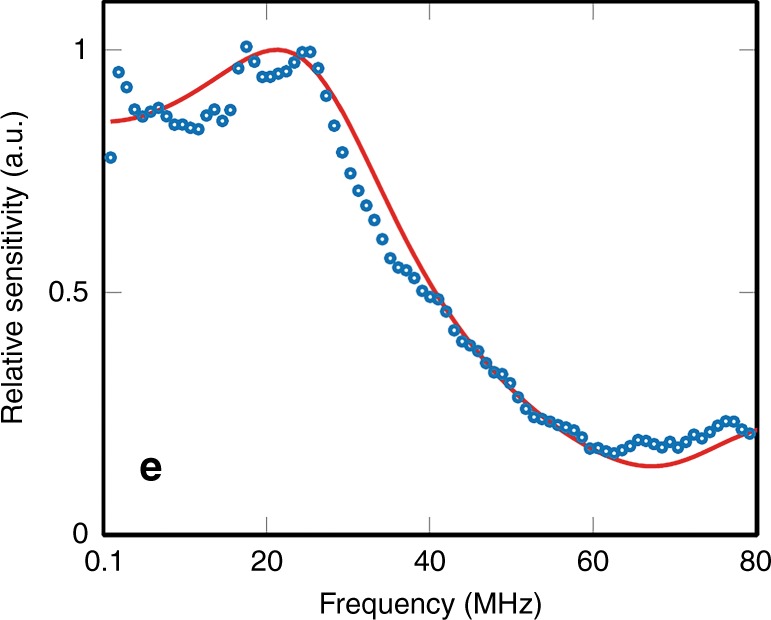
Frequency response of the FP sensor from a single fiber-optic core. Blue dots: measured response, red line: modeled frequency response^31^

The acoustic bandwidth of the sensor was measured using a wideband (1–70 MHz) laser-generated ultrasound source and a calibrated ultrasound sensor of known frequency response^30^. Figure 2 demonstrates that the sensor provides a smooth, well-behaved broadband acoustic frequency response with a -3 dB bandwidth of 34 MHz, a small *λ*/4 resonance at 21 MHz and a *λ*/2 minimum at 67 MHz. These features are characteristic of the rigid-backed configuration that is represented by the fused silica fiber bundle in contact with the polymer-film spacer and is in close agreement with the modeled response^31^. The noise-equivalent pressure (NEP) was measured using a calibrated source^25^. It varies from core to core, depending on the coupling efficiency (Suppl. Fig. S2) and was found to lie in the range 0.5–1.26 kPa (see Suppl. Fig. S4) over a 20 MHz measurement bandwidth.

### Image acquisition and reconstruction

Accurately reconstructing an image in tomography mode requires precise knowledge of the locations of the fiber cores at the distal end of the bundle. The cores are arranged in a hexagonal lattice (as it offers the highest packing density), and the spacing between the cores varies slightly over the entire aperture. To determine the positions of the cores, a rapid high-resolution scan (2 µm step size) is performed to generate a map of the x,y-coordinates of the cores—see Suppl. Fig. S2 for an example of such a scan. To achieve this, the interrogation laser wavelength was first tuned to a region of zero derivative on the FP ITF, where the reflectivity is independent of the FP sensor optical thickness. Then, the interrogation beam was raster-scanned across the input face of the bundle and the power of the reflected light was recorded in the absence of a photoacoustic signal. The reflected light originates predominantly from the distal end of the bundle since the proximal end is wedged; hence, its reflection is not coupled into the single-mode fiber-optic circulator for detection by the photodiode. Therefore, the reflected intensity is highest at locations that correspond to the centers of the cores, thereby enabling their positions to be precisely determined.

To acquire a photoacoustic image, the interrogation laser beam is directed only to the locations of the cores (as determined by the above procedure). The FP sensor is sufficiently sensitive that signal averaging is not required; hence, each photoacoustic signal is acquired with a single laser pulse. The set of photoacoustic signals that are recorded from all cores is interpolated to a uniform rectilinear grid before reconstructing the photoacoustic image using an algorithm based on time reversal^32,33^.

The high-resolution scan used to ascertain the core locations along with the procedure for determining the optimum bias wavelengths revealed that approximately 10% of the cores, mostly around periphery of the bundle, were found to have low SNR, mainly due to defective non-transmitting cores (see Suppl. Fig. S2). Thus, out of the 50,000 cores of the bundle, photoacoustic signals were acquired at approximately only 45,000 cores during a typical scan.

## Results and discussion

### Spatial resolution

**Fig. 3:**
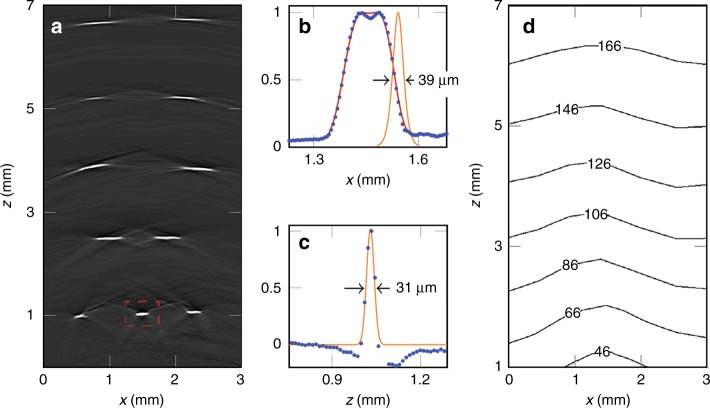
Spatial resolution of the probe. **a** x–z cross-section extracted from the reconstructed 3D photoacoustic image of a multi-layer ribbon phantom showing the individual absorbing ribbons over the probe field of view; the apparent curvature of the features in the top three rows is not an image artifact but reflects the true geometry of the phantom. **b**, **c** Lateral and vertical profiles (blue filled circles), respectively, through the highlighted feature in **a;** The lateral resolution is given by the FWHM of a Gaussian function that is fitted to the derivative of the falling edge (orange line in **b**). The vertical resolution is given by the FWHM of a Gaussian function that is fitted (orange line in **c**) to the vertical profile**. d** A contour plot that shows the variation in the lateral spatial resolution over the x–z plane. Incident fluence: 18 mJ/cm^2^

The spatial resolution of the probe was evaluated by imaging a target that comprised five rows of highly absorbing black polymer ribbons that were immersed in water. A 3D image with a cylindrical field of view of 3.5 × 7 mm was reconstructed from the recorded photoacoustic signals and from this, a 2D vertical (x–z) slice was extracted (Fig. 3a). The lateral spatial resolution was determined by calculating the spatial derivative of the edge response of the reconstructed ribbon features in this slice (Fig. 3b). The contour plot in Fig. 3d shows the variation in lateral spatial resolution over the x–z plane. This demonstrates that the lateral resolution ranges from 40 µm at a depth of 1 mm to 175 µm at a depth of 7 mm. This variation arises because the solid angle that is subtended by the absorbers to the FP sensor plane decreases as the depth increases. Consequently, the effective angular detection aperture decreases with increasing depth, thereby resulting in blurring due to the limited-view problem^34,35^. The axial resolution is 31 µm (Fig. 3c) and was found to be largely invariant over the entire field of view.

### Three-dimensional images of phantoms

**Fig. 4:**
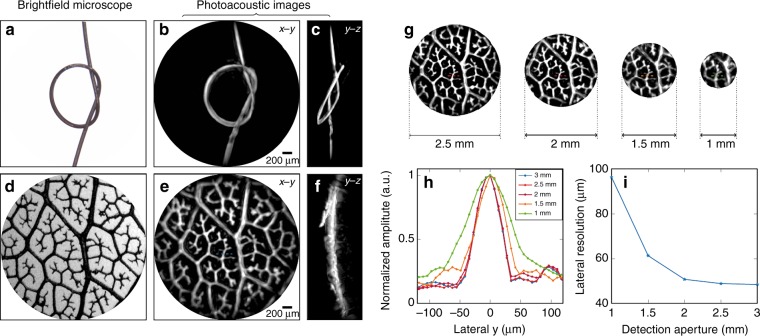
High-resolution 3D photoacoustic images of phantoms. **a** A bright-field microscope image (3 mm across) of a 100 µm diameter synthetic hair knot. **b**, **c** Reconstructed photoacoustic images of the synthetic hair knot shown as x–y and y–z MIPs, respectively. **d** A bright-field microscope image of a leaf skeleton phantom, **e**, **f** Reconstructed photoacoustic images of the leaf phantom shown as x–y and y–z MIPs, respectively. **g** Reconstructed photoacoustic images of the leaf phantom for four detection aperture diameters: 2.5, 2, 1.5, and 1 mm. **h** Profiles of a feature (indicated by horizontal red dotted lines) from the leaf phantom images in **g** for each detection aperture. **i** A plot of the lateral spatial resolution (evaluated by taking the FWHM width of a Gaussian function that is fitted to the profiles in **h**) as a function of the detection aperture. Incident fluence: 18 mJ/cm^2^

To demonstrate the ability of the probe to provide high-resolution volumetric images of absorbing objects of arbitrary 3D geometry, two phantoms that were composed of well-defined absorbing structures, immersed in water and positioned 0.75 mm away from the FP sensor were imaged. One of these phantoms was a synthetic hair of approximately 100 µm diameter that was tied into a knot (Fig. 4a). A 3D image was reconstructed from the detected photoacoustic signals. Figure 4b, c show maximum intensity projections (MIPs) in the x–y and y–z planes, respectively, that were obtained from the reconstructed 3D image data set. Comparison with the bright-field microscope image in Fig. 4a demonstrates that the reconstructed photoacoustic image provides an excellent representation of the target. An animated volume-rendered image is also available online (Media 1), which further illustrates the 3D nature of the image.

The second phantom was a leaf skeleton phantom that was dyed in black India ink, which provides an absorbing structure that resembles a vascular network (Fig. 4d). From the reconstructed 3D photoacoustic image data set, MIPs in the x–y and y–z planes are shown in Fig. 4e, f and an animated volume-rendered image is available online (Media 2). Excellent correspondence between the bright-field microscope image in Fig. 4d and the photoacoustic image in Fig. 4e is evident with the intricate vein structures of the leaf skeleton phantom, some of them as fine as 40 µm, being accurately reproduced with high-resolution and spatial fidelity.

The images of the skeleton leaf phantom in Fig. 4e, f were obtained by recording the PA signals over the full 3.2 mm diameter aperture of the fiber bundle. In principle, smaller-diameter fiber bundles could be used for applications that require a higher degree of miniaturization. However, this would result in a reduced detection aperture, thereby compromising image resolution and fidelity due to the limited-view problem. To explore the extent of this image degradation, the recorded photoacoustic signal data set that was acquired using the leaf phantom was truncated to synthesize various detection apertures. Then, 3D photoacoustic images were reconstructed from the truncated measured data. Fig. 4g shows the x–y MIP photoacoustic images that were formed from these data for 2.5, 2, 1.5, and 1 mm diameter apertures, respectively. The fine structure of the leaf skeleton phantom appears to have been accurately reproduced for all of the apertures, although the resolution has decreased with the aperture size. To assess the latter, line profiles were acquired via a feature in the center of the field of view of all the photoacoustic images, as shown in Fig. 4h. The lateral spatial resolution was measured as the FWHM width of the Gaussian fit to the profiles. Figure 4i shows that the resolution remains almost constant for apertures that are greater than 2 mm but degrades for smaller apertures, particularly those less than 1.5 mm. Nevertheless, surprisingly high image quality is achievable even for a very small detection aperture. This may be a consequence of the wide bandwidth of the sensor and the very fine spatial sampling that is achieved by addressing the FP sensor with a fiber bundle. These results suggest that it may be possible to realize a probe that is as small as 1 mm in diameter that achieves usefully high image resolution for applications that require an ultra-fine 3D imaging device.

### Three-dimensional images of microvascular anatomy

**Fig. 5:**
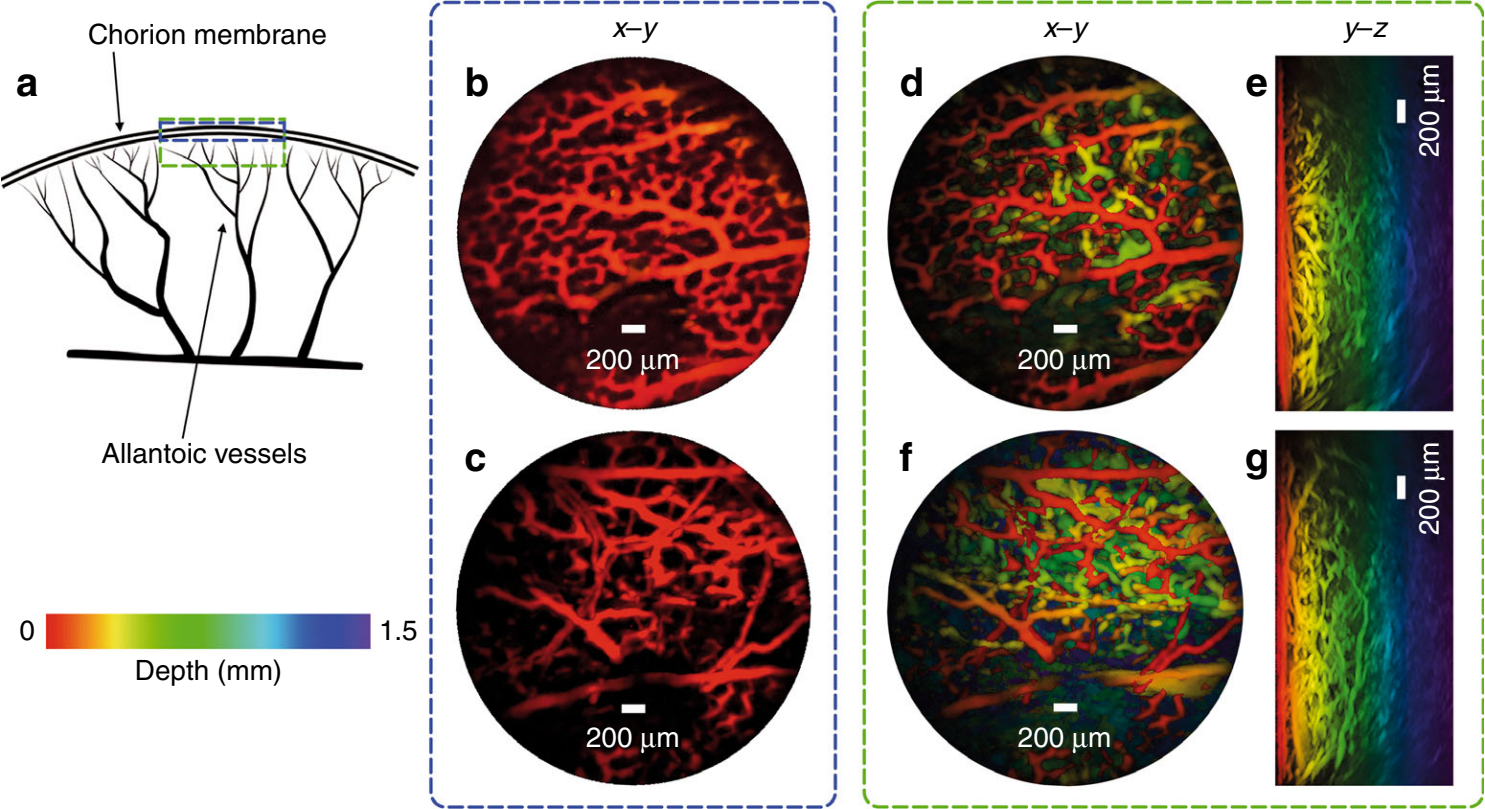
Photoacoustic images of an ex vivo duck embryo. **a** A schematic diagram of an avian embryonic vasculature. **b**, **c** x–y MIPs for depth range *z* = 0–200 µm of two regions of the same embryo, which show the microvascular anatomy of the chorioallantoic membrane. **d**, **f** x–y MIPs for the depth range *z* = 0–1.5 mm for the same two regions as in **b** and **c**, respectively. **e**, **g** y–z MIPs. Excitation laser wavelength: 590 nm, PRF: 30 Hz, and incident fluence: 15 mJ/cm^2^

To demonstrate the ability of the probe to image a biologically realistic target, photoacoustic images of the microvasculature in an ex vivo duck embryo and mouse skin were acquired. The excitation wavelength was 590 nm and the pulse energy at the tip of the probe was 15 mJ/cm^2^, which is below the maximum permissible exposure for skin^36^. Figure 5b–g shows x–y and y–z MIPs of the duck embryo 3D image data set.

The fine capillary network of the chorioallantoic membrane (CAM) that surrounds the avian embryo^37^ is clearly visualized, with vessels with diameters as small as approximately 50 µm being visible. The microvascular anatomy is visible only to a depth of 1 mm, which is due in part by the high absorption of hemoglobin at 590 nm and the significantly higher scattering coefficient in yolk compared with other biological tissues.

**Fig. 6:**
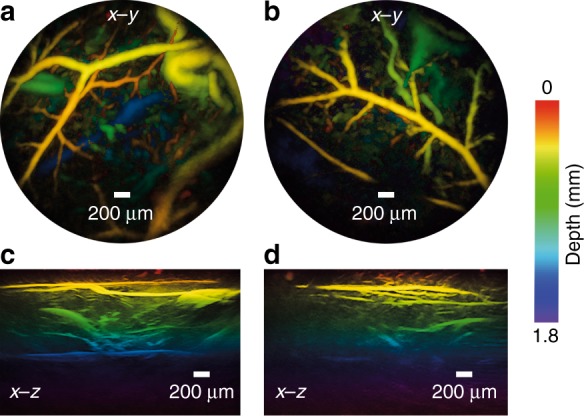
Photoacoustic images of mouse abdominal skin microvasculature. **a**, **b** x–y MIPs for the depth range *z* = 0–2 mm of two regions, **c**, **d** Corresponding vertical x–z MIPs of the same regions as in **a**, **b**. Excitation laser wavelength: 590 nm, PRF: 30 Hz, and incident fluence: 15 mJ/cm^2^

To image the microvasculature in the mouse skin, a five-week-old BALB/C mouse that weighed 15 g was sacrificed by isoflurane overdose and its abdominal side was depilated using commercially available hair-removal cream. Deionized water was used to acoustically couple the mouse abdominal skin with the distal end of the probe. x–y and x–z MIPs corresponding to two distinct areas of the abdomen are shown in Fig. 6. At the 590 nm excitation wavelength, the dominant absorber in the ex vivo tissue is deoxyhemoglobin and the photoacoustic images can distinguish the blood vessels from the surrounding tissue to a depth of approximately 2 mm. The images show the blood vessels, which range in diameter from 53 µm to 180 µm, in the dermis and hypodermis layers of the abdominal skin.

## Conclusions

A miniaturized 3D endoscopic photoacoustic imaging probe that provides a forward-viewing capability has been demonstrated for the first time. The forward-viewing ability is particularly relevant to guiding minimally invasive interventions, especially those that involve needle- or laser-based procedures^22,23,38–40,49^ where it is often desirable to “look ahead” of the insertion direction. By contrast, most previous endoscopic photoacoustic imaging devices provide a 90 degree side-viewing capability, which is often non -optimal for these applications. The forward-viewing capability is made possible by the transparent nature of the FP ultrasound sensor, which allows it to be aligned coaxially with the fiber bundle and the excitation laser pulses to be transmitted through it. This feature of the sensor also enables a high degree of minaturisation to be achieved (limited only by the diameter of the fiber bundle) since it avoids the need to laterally offset the detector from the delivery fiber. In this study, a probe with a diameter of 3.2 mm, which is sufficiently small to be inserted into the working channel of a standard endoscope or laparoscope, was demonstrated. However, since the FP sensor can be deposited on to a substrate of arbitrarily small dimensions, smaller probes with a diameter of 1 mm or less could be fabricated for endovascular use, delivery via biopsy needle or other situations that require an ultra-fine imaging probe. As illustrated in Fig. 4g, image resolution and fidelity are surprisingly high for superficial features, even for a detection aperture as small as 1 mm; however, image quality will degrade with depth and this degradation may be more rapid for small apertures. While a rigid fiber bundle was used in the current study, a flexible fiber bundle could be used to image anatomical sites that require access via tortuous routes using flexible endoscopes^41^.

The probe provides high spatial resolution and excellent image quality, as evidenced by Figs. 4–6. This is a consequence of several factors: First, the high packing density of the fiber bundle enables very fine spatial sampling, thereby satisfying the spatial Nyquist criterion at frequencies in the tens-of-MHz range as required for high-resolution photoacoustic imaging. Second, the small element size (12 µm) yields low directional sensitivity, which is an important additional requirement for an accurate image reconstruction. Third, the sensor provides the necessary broadband, well-behaved frequency response for faithfully recording the wideband acoustic frequency spectrum of photoacoustic waves. Achieving a similar level of spatial sampling and bandwidth using conventional piezoelectric-based detection methods would be extremely challenging. The challenge in terms of spatial sampling becomes most apparent if the FP sensor at the tip of the fiber bundle is regarded as a 2D array of 50,000 (the number of fiber cores) ultrasound receivers, each of 12 µm element diameter (the fiber core diameter) that are distributed within the 3.2 mm diameter footprint of the probe. Even if a similarly high-density piezoelectric array could be fabricated, it would be difficult to achieve sufficient sensitivity with similar element diameters (i.e., 12 µm). However, one limitation of the current embodiment is that each “element” of the FP sensor array is sequentially addressed. In this study, only a single interrogation beam was used and the maximum PRF of the excitation laser was 30 Hz, which resulted in an acquisition time of approximately 25 min for the images in Figs. 5 and 6. Although this is impracticably long for clinical use, there is significant scope for reducing the acquisition time. It has previously been shown using a free-space FP sensor-based scanner that 3D images can be acquired in 10 seconds^42,43^ and 2D images at video frame rates^42^ by increasing the excitation laser PRF to 200 Hz and parallelizing the sensor read-out. The latter can be achieved by scanning multiple beams^42,43^ or using full-field illumination^44^. Even faster acquisition may be possible by using sub-sampling and compressed sensing techniques^45,46^, thereby offering the prospect of real-time 3D imaging.

In this feasibility study, the tissue penetration depth was limited to approximately 2 mm. This is in part due to the relatively low finesse of the FP sensor, which is a consequence of the low mirror reflectivities that were used. By increasing the reflectivities of the mirrors and optimizing their ratio to account for the spatial aperturing that is imposed by the core^28^, it is anticipated that comparable sensitivities to previously demonstrated free-space interrogated FP sensors^25^ will be achievable. This would enable in vivo tissue penetration depths of approximately 10 mm to be achieved^26,47^. In addition, further increases in penetration depth may be possible via the use of microresonator-based sensors, which provide order-of-magnitude-higher sensitivity than the planar FP etalon sensor^48^. There is also the potential to increase the spatial resolution, albeit at the cost of depth, by increasing the sensor bandwidth if required. The bandwidth of the FP sensor that was used in this study was 34 MHz. However, by reducing the thickness of the polymer-film spacer, this can be increased to 100 MHz or higher.

In addition to high photoacoustic image quality and small probe size, the proposed technology offers simple and inexpensive sensor fabrication, no moving parts at the distal end and improved safety for endoscopic use due to the absence of conductive components and biocompatibility. Immunity to EMI is afforded by the inert dielectric nature of the probe materials, which also suggests that it is likely to be compatible with MRI. Moreover, it offers ease of integration with optical imaging modalities. For example, the FP sensor is transparent to visible wavelengths; hence, it is possible to perform simultaneous video endoscopy with the same fiber bundle. Similarly, it can potentially be combined with OCT^11^, confocal endomicroscopy and other scanning optical imaging techniques.

In summary, this work sets the scene for a new class of high-resolution endoscopic photoacoustic devices that offer a forward-viewing capability that could find application for guiding laparoscopic procedures, fetal surgery and other interventional procedures.

## Electronic supplementary material

PA volume-rendered image of a synthetic hair knot phantom

PA volume-rendered image of a leaf skeleton phantom

Supplementary Information
