# Supplementary material for: All-optical forward-viewing photoacoustic probe for high-resolution 3D endoscopy
Source: Light Sci Appl. 2018 Oct 10;7:75. doi: 10.1038/s41377-018-0070-5 (PMC6177463; doi:10.1038/s41377-018-0070-5)
Supplement: Supplementary file 3 — Supplementary Information [file 41377_2018_70_MOESM3_ESM.docx]

**Supplementary Information**

**All-optical forward viewing photoacoustic probe for high resolution 3D endoscopy**

Rehman Ansari^1,2^, Edward Z. Zhang^1,2^, Adrien E. Desjardins^1,2^ and Paul C. Beard^1,2^

^1^Department of Medical Physics and Biomedical Engineering, University College London, Gower Street, London WC1E 6BT, UK

^2^Wellcome/EPSRC Centre for Interventional and Surgical Sciences, University College London, Charles Bell House, 67-73 Riding House Street, London, W1W 7EJ, UK


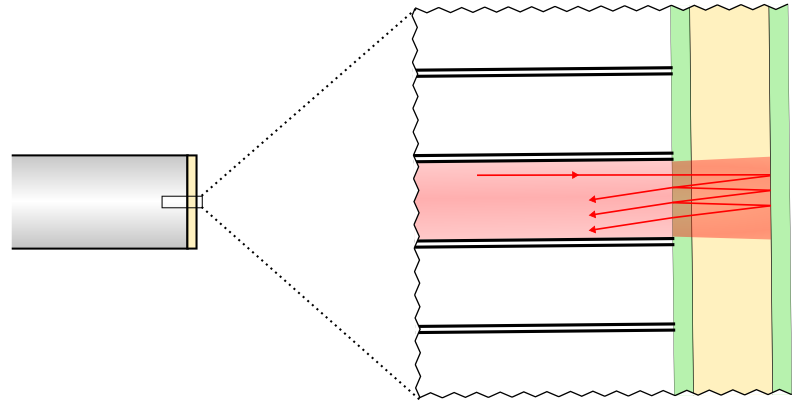


Fibre bundle

Parylene spacer

Mirrors

**Figure S1: FP sensor transduction mechanism:** FP sensor structure illuminated by a single core of the fibre bundle. The sensor comprises a pair of mirrors separated by a 15-µm-thick Parylene spacer, thus forming a FP interferometer. The mirrors of the interferometer are dichroic: highly reflective in the sensor interrogation wavelength range (1,400–1,600 nm) but transparent to the excitation laser wavelengths used (580–1250 nm) – the latter allows the pulsed excitation laser beam to be transmitted through the sensor into the adjacent tissue. The sensor is interrogated using a continuous wave (CW) laser beam that is coupled into the fibre core. The interrogation light emerges from the core and is reflected back and forth within the Parylene space (only two reflections are shown in the figure). Photoacoustic waves generated in the tissue are incident on the sensor producing a strain in the Parylene spacer. This results in a change in the optical thickness of the spacer, and thus the phase between the optical fields reflected from the two mirrors. Providing the interrogation laser wavelength is tuned to the peak derivative of the interferometer transfer function, this phase change is linearly converted to a corresponding temporal modulation of the reflected optical power and detected by a photodiode.

**Figure S2.** **Reflectance map of the fibre bundle showing all 50,000 cores.** The map was acquired by scanning the proximal end-face of the bundle with the focused interrogation laser beam. The interrogation laser wavelength was chosen to be significantly different from the bias wavelength in order to avoid reflectivity changes due to variations in the optical thickness of the FP sensor located at the distal end of the bundle. The intensity distribution in the map predominantly represents the light reflected from the FP sensor since the proximal end of the bundle is wedged so that its reflection is not coupled into the single mode fibre-optic circulator (see fig 1) and detected by the photodiode. The map shows that the fibre-optic cores in the centre of the scanned area are clearly resolved and have higher reflected intensity. By contrast, at the periphery, the fibre-optic cores have lower reflected intensity due to the reduced coupling efficiency arising from off-axis aberrations of the scanning lens. This spatial variation in coupling efficiency contributes, in part, to the variations in sensitivity shown in the NEP histogram in figure S4. The map above also reveals some regions where the cores are defective. For this reason, photoacoustic signals were acquired at approximately 90% of the total number of cores (typically 45,000) during a typical scan.


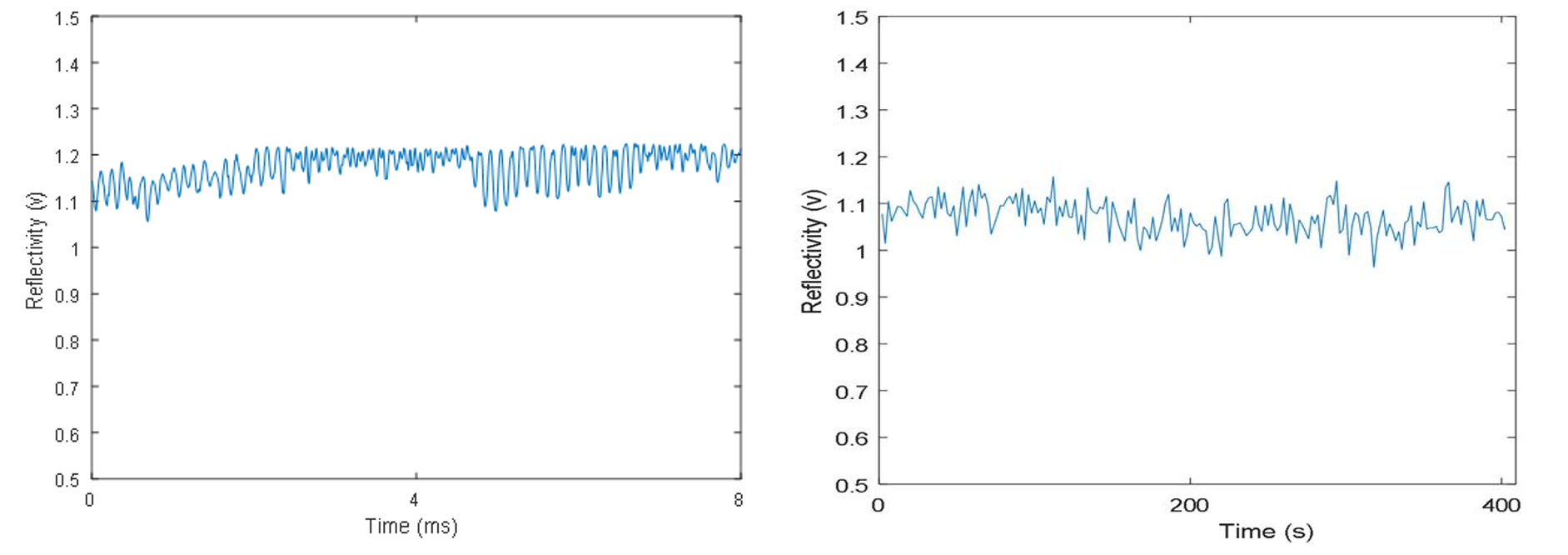


1. (b)

**Figure S3:** **Measurements of coupling efficiency stability and repeatability**. (a) Continuous measurement of reflectivity over a period of 8ms obtained by monitoring the light reflected from a single core. (b) Measurement of the reflectivity of a single core obtained by repeatedly scanning the fibre-bundle end face and returning to the same core over a period of 400 seconds.

Over the duration of a typical image acquisition, the coupling efficiency appears to be quite stable. Figure S3(a) shows that the light reflected from a single core over a period of 8ms (which is comparable to the dwell time at each core during the acquisition of a single photoacoustic signal) is stable to within approximately 10% with the oscillations being due to jitter on the galvanometers. Figure S3(b)) shows the ability of the scanner to repeatedly to locate a particular core in successive raster scans over a period of 400s. Again the variation is approximately 10%. These results show that variations in coupling efficiency over the timescale of a typical image acquisition are modest.

**Figure S4:** **NEP histogram for the fibre bundle interrogated FP sensor over a 20 MHz measurement bandwidth**. The vertical axis N represents the percentage of the total number of fibre-optic cores (~45,000). Variation in the NEP is estimated by fitting a Gaussian function to the histogram and the FWHM range is 0.5 kPa to 1.26 kPa.
